# A case report of an atypical severe case of skin picking disorder managed by a multidisciplinary team

**DOI:** 10.1186/s12888-024-05712-4

**Published:** 2024-03-28

**Authors:** Marissa A. LeBlanc, Mary-Ann Hudec, Lutz Weise

**Affiliations:** 1 Department of Psychiatry, Dalhousie University, Abbie J. Lane Building, 6509-5909 Veteran’s Memorial Lane, Halifax, NS B3H 2E2 Canada; 2 Nova Scotia Health, Halifax, NS Canada; 3 Division of Neurosurgery, Dalhousie University, Halifax, NS Canada

**Keywords:** Case report, Skin picking disorder, Psychiatry, Multidisciplinary

## Abstract

**Background:**

While skin picking disorder remains relatively common, it often does not present to psychiatry until significant morbidity or comorbidities are reached. It is described as recurrent picking of skin leading to skin lesions, with repeated attempts to decrease or stop skin picking. It is also often associated with significant distress or functional impairment. There has been limited research in this specific disorder and treatment efficacy has often been poor in severe cases. For various reasons, only a small amount of patients with this disorder present to care, and often to a multidisciplinary team prior to psychiatry.

**Case presentation:**

This is a case presentation of a 44 year old male with a complex past psychiatric history, ultimately untreated for an underlying skin picking disorder. He presented for urgent medical care following a self-inflicted wound through the central frontal bone and dura over the course of 2 years. He was treated with current psychiatric evidence based medicine, including an SSRI, antipsychotic augmentation and NAC, along with habit reversal techniques during the admission. He was concurrently managed with the neurosurgery team, initially with a poor prognosis due to the severity of his presentation. He required debriding of the devitalized bone within the adjacent brain to cover the dural defect, IV antibiotics for 6 weeks, and an initial skin graft on his initial admission.

**Conclusions:**

This case in particular highlighted the importance of urgent treatment via a multidisciplinary approach to avoid mortality. It highlights the importance of increasing awareness about the disorder and that treatment with SSRI’s, along with antipsychotic and NAC adjuncts remains the mainstay of acute treatment.

## Background

Skin picking disorder is only a recent new entity in the psychiatric classification system of the Diagnostic and Statistical Manual of Mental Disorder (DSM) 5th edition [1]. It is described as recurrent picking of skin leading to skin lesions, repeated attempts to decrease or stop skin picking, and it is associated with significant distress or functional impairment. It is listed in the section of obsessive–compulsive and related disorders and has significant overlap with other classified disorders such as trichotillomania (hair-pulling disorder).

Historically, there has been limited research in this specific disorder and treatment efficacy has often been poor in severe cases. Ultimately, only a small portion of patients seek help, noted to be due to reasons such as embarrassment, stigma, belief that it is a “bad habit”, or that it is untreatable [2]. In addition, many of the patients initially present to a general practitioner/family physician or a dermatologist before ever being consulted to a psychiatrist [2]. A recent systematic review of treatment options found that current management options included both a behavioural approach (habit reversal or cognitive-behavioural therapy, specifically acceptance-enhanced behaviour) and medication management (selective serotonin reuptake inhibitor [SSRI] or N-acetyl cysteine [NAC] [3]. The severity of skin-picking disorder can range from mild-severe; and not all cases need medication treatment.

Despite a less than comprehensible approach to treatment or presentation of skin picking disorder, it remains a relatively common disorder, with prevalence estimating to range between 1.4 – 5.4% [4]. It may present itself at any age, but most commonly coincides with the onset of puberty during adolescence [5]. It may be triggered by other dermatological conditions such as acne or eczema, but is often multifactorial in terms of triggers and can include stress, anger, anxiety, boredom among others [6]. Most common areas observed for skin picking is the face, followed by hands, fingers, arms and legs.

In more severe cases, if patients are not help-seeking, they likely will be unable to stop picking despite repeated efforts. This may lead to furthering of shame, isolation and ultimately even development of an anxiety disorder or major depressive disorder. The functional impact on these patients should not be underestimated as they may spend an impressive amount of time hiding this behaviour or performing the repetitive behaviour of picking. This can have subsequent psychosocial, personal and professional consequences. Medically, this can also have significant morbidity and mortality, including infections, scaring, and even serious physical disfigurement [7]. Psychiatrically, it is also associated with other comorbid conditions, such as mood and anxiety disorders.

Recent reviews studied the clinical efficacy but also the tolerability of a number of pharmacological agents for treating or reducing skin-picking disorders. This has included Serotonin Selective Reuptake Inhibitors (SSRI’s), lamotrigine, glutamateric agents such as NAC, opioid antagonists such as naltrexone [3, 8] and augmentation strategies. SSRI’s have been identified as the foundation of pharmacotherapy for skin-picking disorder, supported by a randomized controlled trial that suggested NAC should also be considered as a possible option [9]. There was no systematic studies investigating the efficacy and tolerability of augmentation agents, but case studies supported atypical and typical antipsychotic agents in reducing skin picking. Augmentation agents included Olanzapine, Haloperidol and Aripiprazole as potential adjuncts [3].

## Case presentation

This is a case presentation of a 44-year-old male with a significant past psychiatric history from 10 + years that included various diagnoses including schizotypal personality disorder, dependent personality disorder, schizoaffective disorder, schizophrenia, anxiety disorder and skin picking disorder. He was referred to consultation-liaison psychiatry when admitted to the intensive care unit following a self-inflicted wound through the central frontal bone and dura. The wound was inflicted via self picking and eventually with the use of a “butter knife” over the course of 2 years.

Prior to this presentation, he had been followed by a community team with minimal clinical effective treatment, likely due at least in part to unclear diagnoses. Initial admission psychotropic medications included Clomipramine, Quetiapine, Clonazepam, Paroxetine, Risperidone, Pregabalin, and Valproic acid (for a seizure disorder). As patient was intubated on initial assessment, we reviewed his history from clinical notes and gathered collateral (including from family doctor who had continuous contact with patient and family) to help with diagnostic clarity. There was no clear evidence that the patient had any severe persistent mental illness such as schizophrenia or schizoaffective disorder. There was no reported psychosis (hallucinations or delusions), no disorganized behaviour otherwise, and no disorganized speech. Rather, he had a constellation of symptoms that included components of general anxiety and personality disorders. In addition, he had overvalued ideas (i.e. bizarre beliefs related to the need of skin picking), but when further investigated this was more in keeping with longstanding beliefs of his rather than true delusions.

As per DSM-5 criteria, there was recurrent picking of skin leading to skin lesions, repeated attempts to decrease or stop skin picking (patient had reported awareness that this was a problem) and it was associated with significant distress and functional impairment. His skin picking also appeared to be in the context of long-standing anxiety, potentially low intellectual disability (as noted by family doctor and family) and potentially even exacerbated by sub-optimal management of his psychiatric medications over the years. There was unfortunately no formal IQ testing done prior this presentation as these services were not available or accessible to the family.

**Fig. 1:**
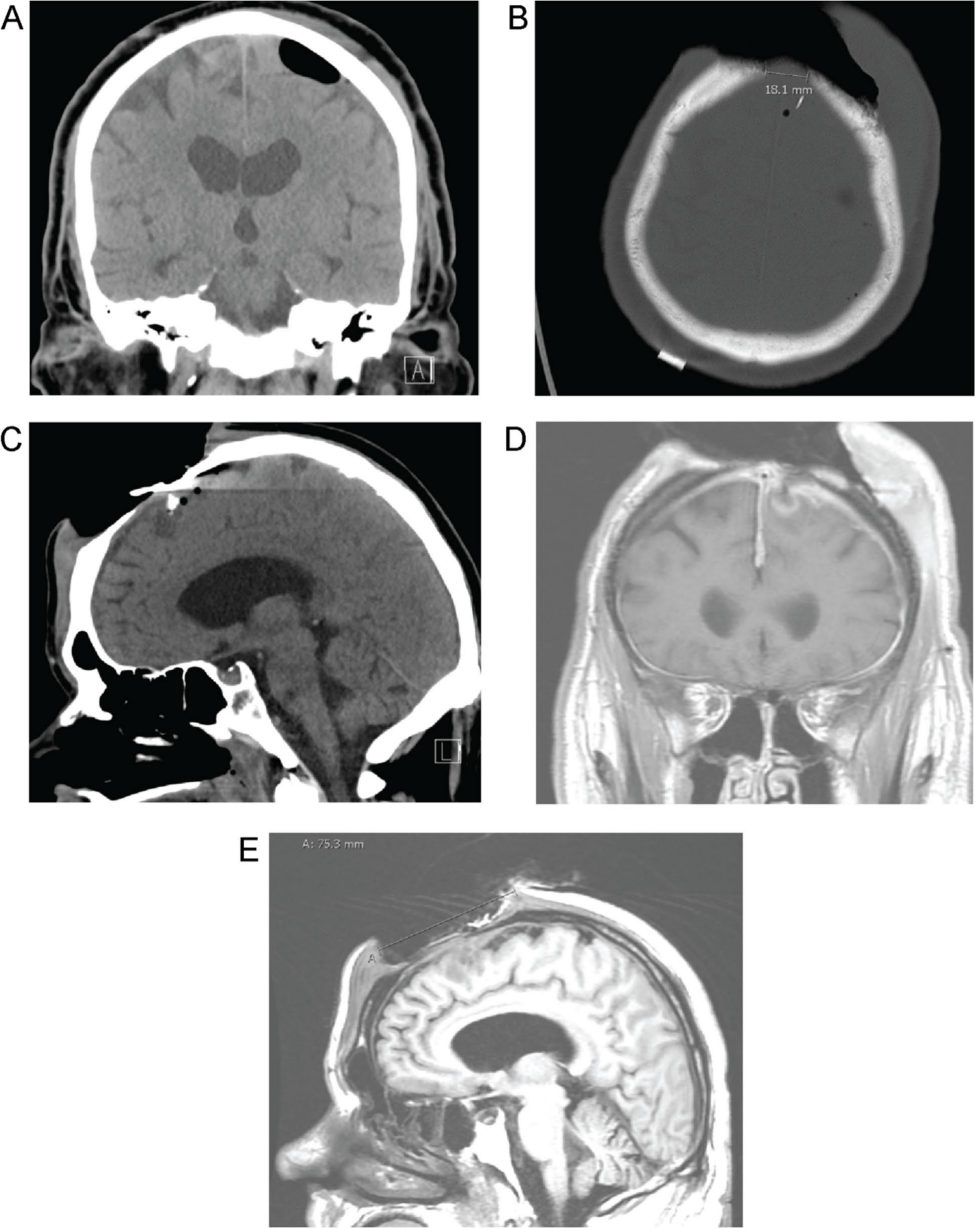
Imaging on initial presentation: **A**: Coronal CT demonstrating left parasagittal intracranial air as a sign of a dural defect; **B**: Axial CT (bone window) demonstrating the bone defect, a bone splinter and the depth of the skin defect; **C**: Sagittal CT again showing the bone splinter entering the frontal lobe as well as the sagittal extent of the scalp and bone defect; **D**: Coronal MRI showing gadolinium enhancement underneath the defect as a sign of encephalitis; **E**: Sagittal MRI demonstrating the extent of brain involvement

The patient was initially admitted by the neurosurgery team as he had eroded a large portion of his scalp, the underlying frontal bone as well as the dura in close proximity to the sagittal sinus, resulting in an epidural abscess and leakage of cerebrospinal fluid (Fig. 1). His initial prognosis was poor – with risks of infection, bleeding and even death due to the risk of exsanguination or thrombosis of his sagittal sinus. In addition to the initial neuro-surgical stabilization, and from a literature review on skin picking disorder, we made the following medication changes on admission: discontinued Quetiapine, Clomipramine, Risperidone, Trazodone, Pregabalin, with a slower taper of Paroxetine over 3–4 weeks. We also initiated Fluoxetine which was titrated to 60 mg, Olanzapine 15 mg and NAC 600 mg qAM and 1200 mg qHS. SSRI, antipsychotic augmentation and NAC all have evidence in treating skin picking disorder [3] as previously discussed.

**Fig. 2:**
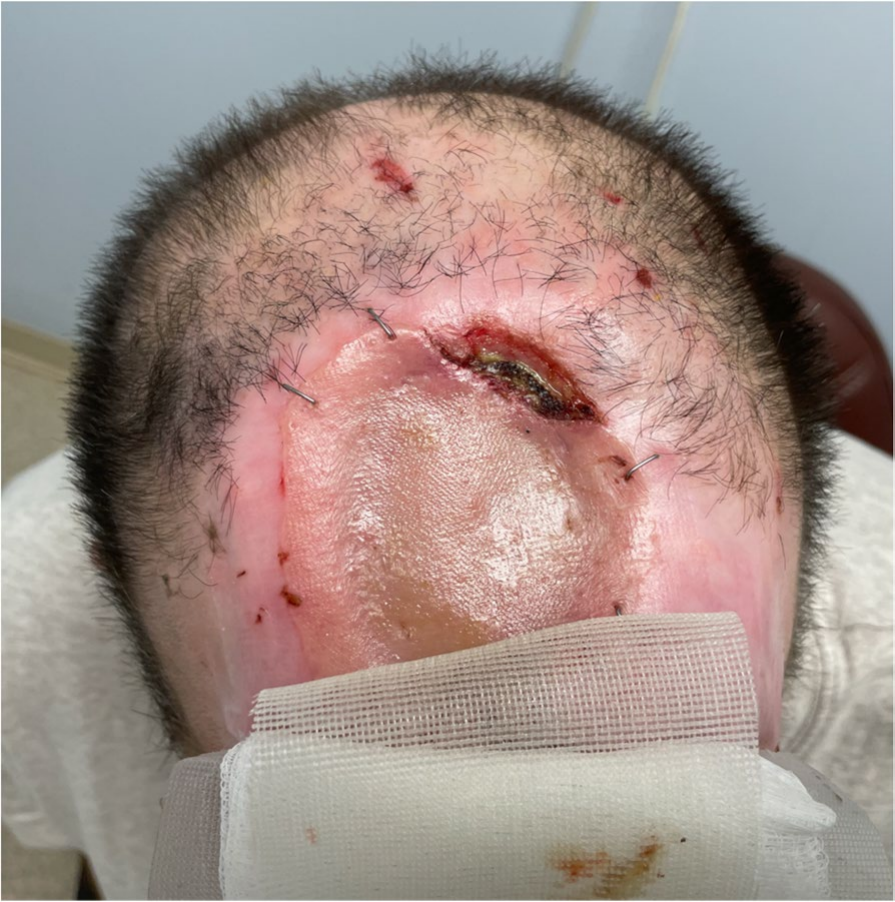
3 months post initial reconstructive surgery showing skin epithelization

After stabilizing the patient in the intensive care unit, a neurosurgical procedure was performed in conjunction with plastic surgery debriding devitalized bone within the adjacent brain and covering the dural defect. Over time, and with the new treatment described, he significantly reduced his skin picking which allowed some healing of the bone. He concurrently had significant improvement in his mental status exam. He became alert, oriented and engaged in his care. He was cooperative and had no disorganized behaviour. His speech was normal. He had no evidence of psychosis, including no delusions and no hallucinations. He denied any beliefs of self-harm. He had insight that his self-inflicted wound was problematic but described an ongoing need to do so. He continued his new medication treatment regimen and started psychotherapy for habit reversal techniques during the admission. He also remained on IV antibiotics for 6 weeks, after which he received an initial skin graft. The skin healed with full epithelialization (Fig. 2). A second plastic reconstructive surgery was scheduled for 6 months. His mental status exam continued to improve during the admission, with no evidence of psychosis and ongoing insight into his skin picking disorder.

## Conclusion

Healthcare providers need to be aware that skin picking disorder can have significant impact on a patient’s life. If presenting primarily to a non-psychiatrist, the treating clinician needs to be aware of when to escalate for further treatment. Diagnosis, education, psychotherapy and potentially pharmacological management may be required. Severe cases should be referred to a psychiatrist specialist service and due to the level of mortality, comorbidity and even mortality that can be associated with this, may even need to be prioritized as urgent.

## Data Availability

The datasets used and/or analysed during the current study available from the corresponding author on reasonable request. All data generated or analysed during this study are included in this published article [and its supplementary information files].

## Abbreviations

DSM: Diagnostic and Statistical Manual of Mental Disorder
SSRI: Selective serotonin reuptake inhibitor
NAC: N-acetyl cysteine
